# Tubercular Ulcer of Tongue in an Elderly Patient Masquerading as a Traumatic Ulcer

**DOI:** 10.1155/2017/8416963

**Published:** 2017-06-28

**Authors:** Ramesh Parajuli, Sushna Maharjan

**Affiliations:** ^1^Department of Otorhinolaryngology, Chitwan Medical College Teaching Hospital, P.O. Box 42, Bharatpur 10, Chitwan, Nepal; ^2^Department of Pathology, Chitwan Medical College Teaching Hospital, P.O. Box 42, Bharatpur 10, Chitwan, Nepal

## Abstract

Tuberculosis is still one of the most prevalent diseases in developing countries like Nepal. However, due to the effectiveness of DOTS therapy, vaccination, and education, the prevalence of tuberculosis has fallen in recent years. Although the pulmonary and extrapulmonary tuberculosis, especially the tubercular lymphadenitis, is still very common in our country, the tuberculosis of oral cavity is an uncommon condition. We present a case of an elderly male who presented with the complaint of nonhealing ulcer of lateral border of tongue for 2-month duration which was masquerading as a traumatic ulcer due to sharp teeth adjacent to the lesion. Deep biopsy was taken under local anesthesia. Histopathological examination revealed that it was tuberculosis. Antitubercular therapy was then started which cured the lesion.

## 1. Introduction

Tuberculosis (TB) is an infectious disease caused by the* Mycobacterium* species of bacteria with* Mycobacterium tuberculosis* being the most common strain. The prevalence of TB is decreasing but it has reemerged as an opportunistic infection in immune-compromised individuals such as those affected with human immunodeficiency virus (HIV) infection especially in the developed countries. However, in developing countries like Nepal it has a high prevalence rate. Although tuberculosis can affect almost every organ in our body, lungs are the most commonly affected organ by the tuberculosis. Extrapulmonary tuberculosis in head and neck regions includes cervical lymphadenitis, TB of larynx, TB of tonsils, TB of salivary glands, tubercular otitis media, and tuberculosis of nose and nasopharynx. Tuberculosis of oral cavity is an uncommon disease with an incidence of 0.5–1.5% [1]. Here we are presenting a case of tubercular ulcer of the tongue in an elderly patient in the absence of pulmonary tuberculosis that was mimicking traumatic ulcer.

## 2. Case Presentation

A 78-year-old male was brought to our department with the complaint of painful, progressive, and nonhealing ulcerated lesion of the tongue for 2 months. He did not give any history of fever, night sweats, cough, decreased appetite, or significant weight loss. There was no history of change in voice or choking. He did not give history of traumatic episode preceding the development of that wound. He denied any history of similar lesions in the past. He was a known case of hypertension taking antihypertensive medications. Besides having hypertension he denied history of other systemic illnesses including pulmonary tuberculosis. He admitted to smoking and drinking alcohol occasionally. The rest of his medical and surgical history was unremarkable. His past dental history was not significant. There was no history of TB in his family members. He was repeatedly treated in a local hospital with topical antiseptics and oral antibiotics (ampicillin plus cloxacillin and metronidazole) and analgesics but the lesion did not subside so he was referred to our hospital for the further management.

**Figure 1:**
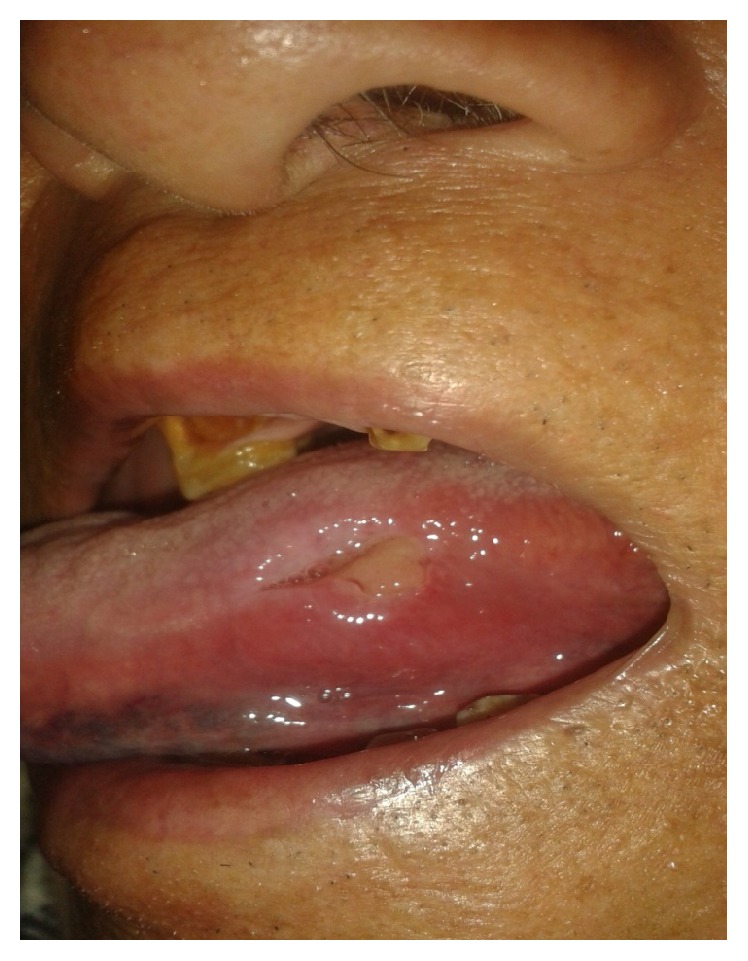
Ulcerated lesion of left lateral border of tongue (photograph was taken after incisional biopsy).

On general examination he was thin built with general conditions being fair, while, on examination of oral cavity, there was an indurated ulcer measuring 1.0 cm × 0.5 cm on the left lateral border of tongue on the anterior two-third part of tongue (Figure 1). It was tender with irregular border. It did not bleed on touch. The remaining part of the tongue was normal in texture and color. Mobility of tongue was normal. It was not deviated to either side on protrusion. He had poor orodental hygiene along with sharp tooth adjacent to the ulcerated lesion although the multiple teeth were absent. There were no palpable cervical lymph nodes and no similar ulcers in any other body parts.

**Figure 2:**
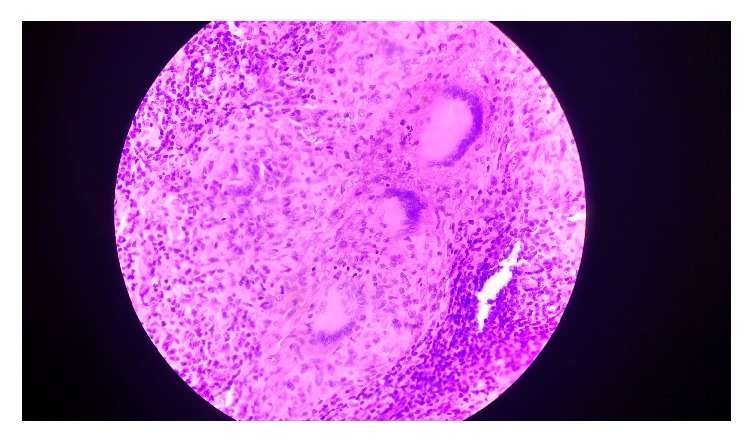
Histopathological picture showing epithelioid cells, Langhans cells, and foci of caseous necrosis.

Provisional diagnosis of traumatic ulcer was made as the possibility of ulcer due to repeated trauma by the sharp tooth could not be ruled out based on the physical examination alone. As we thought it could be due to chronic trauma by the sharp looking teeth although he was unaware of any traumatic episode. Dental consultation was done as the patient had poor oral hygiene along with loss of multiple teeth and the remaining teeth having sharp edges. Dentist did the coronoplasty of adjacent sharp cusp, and since the ulcer presented for more than two months they took incisional biopsy containing ulcerated lesion along with the normal looking margin being taken under local anesthesia and the specimen was sent for histopathological examination (HPE). The patient was also given oral antiseptic gel for local application and given an appointment for oral prophylaxis. Patient then presented to Department of Otorhinolaryngology with the HPE report. Microscopically the lesion revealed the stratified squamous epithelium with granulomatous inflammation containing Langhans type giant cells, epithelioid cells, and foci of caseous necrosis, strongly suggestive of tuberculosis (Figure 2).

Chest X-ray did not reveal any evidence of active or old Koch's infection. Sputum for acid fast bacillus (AFB) of three early morning samples was negative on Ziehl Neelsen stain. Complete blood count was within normal limit. His erythrocyte sedimentation rate (ESR) value was 20 mm/hr. Serum biochemistry and renal function tests were within normal limits. Serological investigation for human immune deficiency virus (HIV) was negative. Mantoux test was not performed as the histopathological findings were strongly suggestive of tuberculosis. An enquiry was made in the family whether they had any symptoms of pulmonary tuberculosis or any other chronic illnesses. No one in the family had any form of TB in the past. They denied symptoms such as chronic cough, night sweats, loss of appetite, or significant weight loss. They were all vaccinated with BCG as per the expanded programme on immunization (EPI) schedule of Nepal. In the absence of any symptoms no further investigations were ordered for them. Mantoux test was not done as well. Because every child gets BCG vaccination at birth in our country, that can yield positive Mantoux result because of the vaccination. As the sputum for AFB was negative in the patient the chance of spread is minimum to others.

**Figure 3:**
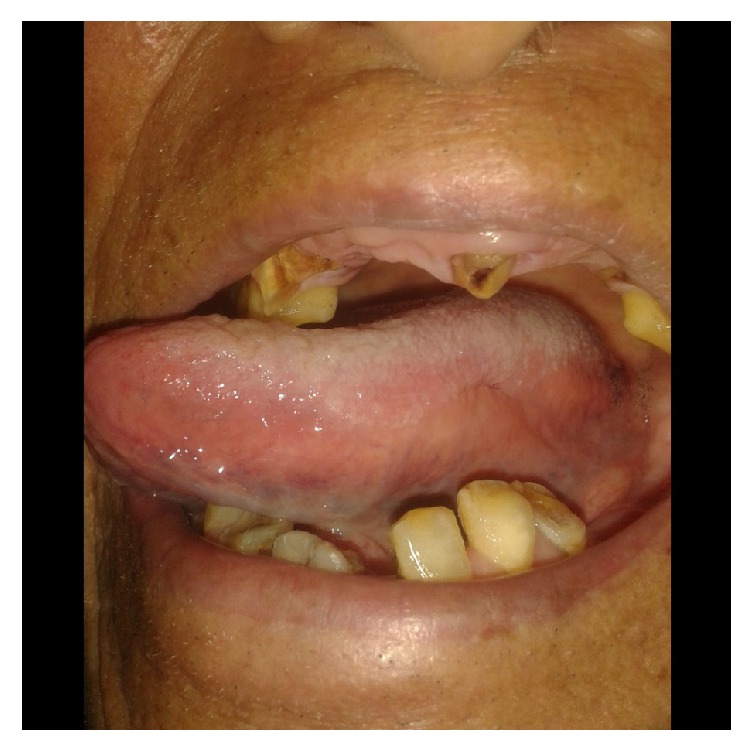
Complete disappearance of ulcerated lesion at the end of 2-month course of antituberculous therapy (ATT).

The patient was referred to directly observed treatment, short course (DOTS) clinic after having histopathological diagnosis of TB. He was then started on antitubercular therapy (ATT). The ulcer gradually started fading once drug therapy was started (ATT). The patient felt symptomatically better after the start of ATT; and the tuberculous ulcer healed completely by the 2 months of ATT containing Isoniazid, Rifampicin, Pyrazinamide, and Ethambutol (Figure 3). The patient was then advised to continue ATT containing Isoniazid and Rifampicin for another 4 months. At the end of total 6 month's ATT course there was no evidence of residual ulcer at the primary site. We emphasized oral hygiene maintenance and advised the patient to follow up at dental outpatient department. As the patient came from a remote place in the hilly region of central Nepal, he did not seek any dental consultation as he was told that he had TB.

## 3. Discussion

Tuberculosis is one of the most prevalent diseases in Nepal. About 45% of the general population of our country have been infected with* Mycobacterium tuberculosis*. But the majority of the infected people are asymptomatic. Primary tuberculosis resulting from initial exposure to mycobacteria is less common than the secondary tuberculosis which is due to reactivation of a latent infection.

Almost any organ in our body can be affected the mycobacteria; lung is the most common site to be involved. However, due to the effectiveness of DOTS therapy, Bacille Calmette-Guerin (BCG) vaccination, and education, the prevalence of tuberculosis has fallen in comparison to the past. Pulmonary tuberculosis can be associated with various forms of extrapulmonary TB. In head and neck regions, TB most commonly presents as cervical lymphadenitis, which accounts for 95% of cases. But the other sites such as the larynx, tonsils, ear and mastoid, nose and nasopharynx, and salivary glands account each for less than 1% of all cases [2].

Investigations such as chest X-ray, ESR, Mantoux test, and sputum for AFB are mandatory in the diagnosis and the treatment of TB, especially the PTB, which is the most common form of TB that is frequently associated with various types of extrapulmonary TB. To diagnose the extrapulmonary TB, there are a number of investigations including mycobacterial stain and culture, examination of body fluid, for example, measurement of adenosine deaminase (ADA), tissue biopsy, polymerase chain reaction (PCR), and immunologic test such as tuberculin skin test. Beside these tests radio-imaging is also a frequently done test.

Although the tuberculosis of oral cavity is rare it should be considered in a differential diagnosis of any longstanding ulcer of tongue. Tuberculosis of oral cavity is rare because of continuous cleaning of oral mucosa by saliva and the resistance of epithelium of the oral mucosa to the mycobacterium. The risk factors responsible for the development of oral tuberculosis include oral mucosal trauma, smoking, and poor oral hygiene. The breach in the mucosal integrity can predispose to the inoculation with the mycobacterium.

Tuberculosis of the oral cavity can be primary or secondary. Secondary tuberculosis to these areas is usually due to pulmonary infections through the contaminated sputum or hematogenous spread. Primary tubercular lesions of oral cavity can occur like TB affecting other organs. Oral lesions of tuberculosis may present as ulcer, nodule, fissure, tuberculoma, or granuloma [3]. Our patient had sharp tooth adjacent to the ulcer at the lateral aspect of the tongue which might have caused chronic irritation favoring the inoculation of the mycobacterium at that site. Oral tuberculosis may occur at any location on the oral mucous membrane, but the tongue is most commonly affected. Other sites include gingival, lips, buccal mucosa, palate, tonsils, and floor of mouth.

The more common differential diagnosis of such ulcerated tongue lesions includes malignant ulcer and traumatic ulcers. Similarly the major aphthous ulcer can cause diagnostic dilemma occasionally. Histologically the granulomatous inflammatory reaction of tubercular ulcer may mimic other granulomatous diseases such as Wegener's granuloma, syphilitic ulcer, actinomycosis, mycotic infections, sarcoidosis, Crohn's disease, cat-scratch disease, foreign-body reactions, tertiary syphilis, and Melkersson-Rosenthal syndrome [4, 5]. Most importantly ulcerated lesion has to be differentiated from carcinoma of tongue because tongue is a one of the commonest sites for malignancy of upper gastrointestinal tract.

Traumatic ulcers usually have the source of trauma evident on examination of oral cavity such as sharp tooth or ill-fitting denture adjacent to the site of ulcer. Whenever there is a diagnostic dilemma the patient should be referred to dentist for the consultation, as it was the case in our patient. Occasionally the malignancy can be confused with tuberculosis although the former has punched-out ulcer with rolled margins and has high chance of locoregional metastasis because the tongue is a highly mobile organ. Tubercular ulcers of tongue usually appear as oval shaped, shallow ulcers with undermined margins which are tender due to exposed nerve endings [4].

Histopathological or cytological examination is mandatory to diagnose the underlying cause of the ulcerated lesion of the tongue. From macroscopic examination alone it is very difficult to differentiate tubercular ulcers of tongue from ulcers due to malignancy or granulomatous diseases. Biopsy of ulcerated lesions of tongue should include deeper tissue as the superficial biopsy may lead to inconclusive diagnosis such as chronic nonspecific inflammation. Thus biopsy may have to be repeated. Once the diagnosis is reached, administration of antitubercular therapy can completely eradicate the tubercular lesions.

When the diagnosis of any form of TB is made in the family, family members should be well educated about the disease, to treat the patient effectively and to prevent the disease in the family and the community to stop its burden on the nation. Droplet inhalation is the most common route for the spread of TB. If the patient is sputum positive then there is greater risk of transmission; hence the patient should be isolated in a well ventilated room until the sputum becomes negative for AFB during treatment with ATT. So the family members should avoid contact with body fluids of the patient and pay more attention to methods of sterilization and disinfection.

## 4. Conclusion

TB is one of the most common infectious diseases in Nepal. Although lung is the most common site, the tuberculosis of oral cavity should be considered in a differential diagnosis of any longstanding ulcer of tongue especially in a developing country like ours. Histopathological examination is the most important diagnostic tool for the diagnosis of ulcerative lesions of the oral cavity. As TB is the infectious disease, all the family members should be educated to prevent the spread of the TB in the family and community. Tuberculosis of tongue does not require complete surgical excision as it can be completely cured with antituberculosis therapy.
